# Staffing and patient-related factors affecting inpatient falls in a psychiatric hospital: a 5-year retrospective matched case–control study

**DOI:** 10.1186/s13033-022-00514-1

**Published:** 2022-01-25

**Authors:** Acharaporn Seeherunwong, Chanya Thunyadee, Wipa Vanishakije, Pavinee Thanabodee-tummajaree

**Affiliations:** 1 Department of Mental Health and Psychiatric Nursing, Faculty of Nursing, Mahidol University, 999 Phuttamonthon 4 Road, Salaya, Nakhon Patthom, 73170 Thailand; 2 Nursing Division, Somdet Chaopraya Institute of Psychiatry, 112, Somdet Chaopraya Rd., Khlong San, Bangkok, 10600 Thailand

**Keywords:** Accidental falls, Case–control studies, Psychiatric hospitals, Risk factors, Staffing

## Abstract

**Background:**

The frequency and severity of falls of psychiatric inpatients in Thailand have not been previously reported. Also, the risk factors of falls have been numerous and complicated. This study aimed to investigate the staffing factors and patient-related factors that increase the likelihood of falls among psychiatric inpatients.

**Methods:**

A five-year retrospective matched case–control study was conducted that selected cases of inpatients who fell and which were reported to the hospital risk management system. Subjects were compared to two control patients from the same hospital (1:2) by age (± 5 years), sex, and admission the same year. The total sample consisted of 240 patients. Univariate and multivariate conditional logistic regression was used to analyze the data.

**Results:**

Of the eighty falls, 86.3% resulted in temporary harm and required intervention. The multivariate model showed that three patient-related factors were associated with increased inpatient falls: (1) having an acute psychotic condition (adjusted odds ratio (AOR) = 4.34; 95% CI 1.45, 13.05; p < 0.05), (2) polypharmacy with more than four types of medicines (AOR = 3.06; 95% CI 1.59, 5.88; p < 0.05), and (3) taking atypical psychiatric drugs (AOR = 2.74; 95% CI 1.35, 5.58; p < 0.05). Staffing for 26–50 patients in the wards was more protective for falls than with 25 or fewer patients (AOR = .17; 95% CI 0.04, 0.72; p < 0.05).

**Conclusions:**

The risk factors found may help assess fall risk and manage the number of patients in psychiatric units. Drug dosage and drug interaction of antipsychotic drugs and cardiovascular agents with other medications and drug administration timing before falls are recommended for further investigation. Also, staff ratio per patient and the proportion of staff mix in psychiatric units need further study to establish adequate levels of surveillance to prevent inpatient falls.

## Introduction

Inpatient falls are an indicator representing quality of care and preventable patient safety issues. Incidence in psychiatric units is 3–4 times falls in general medical units [1]. Compared with nursing home and medical-surgical units, inpatient falls in psychiatry settings have a higher incidence and greater severity [2]. Among all inpatient falls, physical injury is reported thirty percent of the time [3], with four percent serious injury [4].

Falls are a significant cause of morbidity and mortality among psychiatric patients. Falls affect recovery from illness, increase the length of hospital stay, and increase healthcare costs. Moreover, the additional cost of lawsuits from injured patients' families against hospital administrators is significant [3]. In particular, in fall-related severe injuries, the results are loss of function, loss of life, and financial burden [5]. Operational costs for those who fall with serious injury are $ 13,316 more than without a fall, and the length of hospital stay is longer by up to 6.3 days [6]. The psychological impact of falling is fear of a repeated fall, causing patients to restrict their activities.

Factors increasing the risk of falls in a psychiatric setting are complex. The individual level and the organization level or system level affect the fall incidence for psychiatric inpatients. Also, Currie [7] classified the risk factors of falls as intrinsic or extrinsic factors. Intrinsic factors have a physiological origin, while extrinsic factors are those that result from environmental or other hazards. Previous studies identified various conditions as intrinsic factors contributing to psychiatric inpatient falls. A 5-year case–control study reported more physical complaints on the day of the fall, and the prescription of Clonazepam was the most significant predictor of falling [8]. The physical complaints of urinary frequency, generalized weakness, mental status impairment, and dizziness were more frequent among those who fell. Also, the presence of an acute medical condition, more medication, and being prescribed anti-hypertensive medication were differences between those who did or did not fall. Depressed patients aged 20 years and under had a lower risk (relative risk 0.45), and patients between 60 and 70 years old with psychosis had a higher risk (relative risk 2.86) than patients in their respective age groups without these diagnoses [9].

Although psychotropic medications are essential for treating patient symptoms, controversially, such medications were the strongest predictor of falls. A study of psychiatric, geriatric inpatients ≥ 70 years of age taking typical antipsychotic or antidepressants had higher fall incidents [10]. Liperoti et al. [11] also found that elderly institutionalized patients using both atypical and typical antipsychotics had an increased risk of hospitalization for femur fracture than non-users. A prospective matched case–control study [12] reported three out of four factors relating medicine to increased risk of falling. The risk was associated with Parkinson's scores on the extrapyramidal syndrome rating scale, the equivalent dosage of benzodiazepines used, and medication changes within 24 h. These significant factors were associated with falls, a history of falls in the past 6 months, and a lack of history of medical problems.

Most previous studies have shown that patient factors associated with increased falls in psychiatric units are intrinsic factors. Also, the assessment tools and strategies to prevent falls come from intrinsic or patient factors. However, many studies have addressed the importance of system-level or organization-level on reducing fall incidences in psychiatric units. Notably, nursing staff's perception of patient safety in psychiatric inpatient care brings attention to the care environment's crucial role and adequate staffing resources [13, 14].

As Thailand is an upper-middle-income country, health resources are limited in many aspects such as medicine, equipment, financial and staffing. Regarding inpatient falls, time spent providing attention from health workers is crucial; the number of patients per health staff is associated with patients' safety [15]. Evidence of a systematic review has shown that higher nurse-patient ratios are associated with decreased inpatient mortality [16]. In inpatient falls, the association between staffing in terms of the number of register nurse (RN) hours and mixed staff hours was unequivocal. A few exceptional recent studies report an association between RN staffing and the rate of dangerous falls varies by unit type [17]. For example, Lake, Shang, Klaus, Dunton [18] showed a negative association between RN staffing levels and fall rates in intensive care units (ICUs).

Conversely, the level of non-nurse staffing, both Licensed Practical Nurses and assistant nurses, showed a positive association with fall rates in non-ICUs. As in psychiatric units, evidence about staffing factors and fall rates is rare. This current study considers mental health service delivery system in term of human resources including nursing staff and unlicensed nurse assistants in the team to patient ratio and proportion of skill mix to be an extrinsic factor related to falls. Intrinsic factors are included in this current study as well. This study aims to explore fall characteristics and investigate the patient-related and staffing factors that increase the likelihood of falls among psychiatric inpatients. Our results may lead to a better understanding of the multiple factors affecting inpatient falls, and it provides evidence for policy decisions on patient safety care in psychiatric hospitals.

## Methods

A retrospective matched case–control study of psychiatric inpatients matched one patient who had fallen with two control patients who had not by age, sex, and admission year. The study was conducted at a psychiatric hospital located in Bangkok, Thailand.

### Definitions

In this study, falls were defined as an event resulting in a person coming to rest inadvertently on the ground or floor or other lower level. Falls may be fatal or non-fatal and exclude those due to assault and self-harm, though most are non-fatal [19]. Intentional falls in which a patient intentionally descends to the floor were also excluded [20]. Incidents of falls reported to the hospital risk management system were considered cases of falls in this study. The impact of falls was also categorized into five levels of harm (Level E to I), based on the National Coordinating Council for Medication Error Reporting and Prevention classification system [21]. The description of each level is: Level E = resulted in temporary harm to the patient and required intervention, F = resulted in temporary harm to the patient and required initial or prolonged hospitalization, G = resulted in permanent patient harm, H = required intervention necessary to sustain life, and I = resulted in the patient's death. However, in this study, the levels of harm was obtained ranged from level E to Level G.

### Sample size

The sample size was determined using Stata 17.0 to test of association in a 1:2 matched case–control study using conditional logistic regression at a 5% significance level (α), to yield a statistical power (1-β) of 80%. In a previous study, fall incidence in a state psychiatric hospital 0.041 [22] and the odds ratio of exposure to Clonazepam (a benzodiazepine medication) in cases relative to control = 4.05 [8]. Seventy-four cases of falls and 148-controls were required. Assuming 80% of staff reported complete information for both patients and controls, 89 potential cases were sought. According to the 5-year case reports of fall incidences of the hospital, there were 120 cases. However, researchers could find complete information for only 80 patients, accounting for 66.66% of total cases. Therefore, 80 cases of inpatients who fell and 160 inpatients who never fell were included in the analysis.

### Setting

The study was conducted at a psychiatric hospital, governed by the Department of Mental Health, Ministry of Public Health. The hospital is located in Bangkok and was established in 1889. The hospital is a training institute in mental health, psychiatry, and neuropsychiatry for health professionals. The inpatient service has 800 beds, divided into four 15- to 20-bedded private wards, four 60- to 80-bedded ordinary wards, and two 50- to 60-bedded wards for elderly patients. Also, the hospital has a 12 bed-pre ICU ward. The pre-ICU ward was established in 2012 for patients who expressed severe violence toward themselves and others. Hospitals developed criteria to prevent falls by classifying risk into three levels: severe, moderate, and mild. The fall risk levels are evaluated at the day of admission and later by items including (1) history of falling within 1 month, (2) history of any of five illnesses: epilepsy, hypertension, diabetes, cerebrovascular accident (CVA), or osteoarthritis, (3) impaired self-help abilities or disability, (4) a condition of imbalance, such as lack of nutrient or electrolyte imbalance, (5) age 60 years or over, (6) behavior of rapid movement and lack of caution, (7) use of 3 or more drug combined at the same time, (8) use of the drugs chlorpromazine, clonazepam, or clozapine. Each risk level's recommended action was as follows: The group at risk of serious fall was assigned 1:1 staff care with limited behavior as needed and provided supportive devices. Moderately and mild vulnerable fall groups were assigned in-sight, close care staff.

### Data

Cases were defined as psychiatric inpatients aged 18 years or older, having a documented fall reported to the hospital's risk management center between January 1st, 2011 and December 31st, 2014. If a patient fell more than once during admission or another admission in the data collection period, only the first fall on admission was used. Cases were matched to two control patients from the same hospital (1:2) by age (± 5 years), sex, and admission year. The reason for dual controls per case was the rarity of reported cases of falls in the hospital. Each control patient was recruited by purposive sampling based on the match-criteria.

Data on characteristics of falls were obtained from the hospital incident reports for the risk management system. The fall incidents data included year, day of the week (Monday to Sunday), time of the day, type of the ward (specialized, ordinary, and pre-ICU), and level of injury impact. Demographic and clinical data (patient-related factors), were collected from case and control subjects from the patients' history folders. Staffing-related data were obtained from nursing administrator records of the hospital. All data were extracted and recorded by a trained nursing staff member who has worked for 20 years at the hospital. The record form for data collection has three parts. Part 1 is demographic data, comprised of sex, age, marital status, education level, history of falls, consciousness before the fall occurred, and injury level from the fall. Part 2 included clinical data consisting of psychiatric and other diagnoses, all medications received within the previous 24 h, the number of drugs prescribed, and the duration from admission to the fall occurring. Part 3 documented staffing data and comprised the number of nurses and unlicensed nurse assistants (ULNs) on duty, number of patients in the shift, and duration of on-duty hours, both for nurses and ULNs.

The nurse-to-patient ratio was calculated as a staff-related factor by dividing the total number of patients on the ward by the total number of registered nurses during the shift. For analysis, we categorized the nurse-to-patient ratio into three levels: one nurse per 1–15 patients, one nurse per 16–30 patients, and one nurse per 31–45 patients. A score for mixed skills was calculated by summing the working hours of all nurses on the shift divided by the summed total of the working hours of ULNs on the shift. In this study, proportions of mixed skills were calculated to be four values: 0.33, 0.50, 0.67, or 1.00. The number of patients staying in the unit was categorized into three groups: equal or lower than 25, 26–50, and more than 50 patients. The number of nurses and number of ULNs on duty was categorized into two groups: at least one staff member working on the shift, one nurse and above, two ULNs and above, respectively.

### Ethics

This study was reviewed and approved by the Faculty of Nursing Institutional Review Board (COA No. IRB-NS 2015/282.3004) and Review Board committees of the psychiatric hospital. The data history of fall cases and the control group were accessed and recorded in the data collecting form by hospital staff trained to collect data from inpatient folders.

### Statistical analyses

Descriptive statistics were reported for fall incident characteristics and demographic data of the case and control group. Since the fall cases and those who did not fall were match pairs (1:2), comparisons between falls and controls, categorical variables were tested by McNemar test, continuous variables with normal distribution were tested by paired t-test. We used univariate and multivariate conditional (fixed-effects) logistic regression with 95% confidence intervals (CI) to evaluate the association of patient-related factors and staff factors with the risk of fall, displayed as odds ratios. Regarding univariate conditional logistic regression analysis, any significant variable (p ≤ 0.15) related to fall was considered for their possible inclusion in the multivariate logistic regression model. A previous study supported a cut-off value of 0.15 for potential variables contributing to the final model [23]. The final multivariate logistic regression model was selected after assessing the multivariate models' adequacy by Akaike's information criteria (AIC) and Hosmer–Lemeshow goodness-of-fit test. The model with the lowest AIC value compared to other models was selected. Potential interaction between covariates in the model were checked before use in the final model. A *p* < 0.05 was considered statistically significant. All *p* values were two-tailed. The statistical software used for all analyses was Stata17.0

## Results

Of the 80 patients who had fallen and the 160 who had not, 67.5% were male in both groups. The mean age of those who had fallen was 47.37 years, ± SD = 12.95 (range 20–70), and was 47.01 years, ± SD = 13.19 (range 18–70) for controls. The number of patients whose marital status was single, separated, or divorced was 2–3 times greater than those who lived as a couple in both groups. Patients who had had primary school education (6 years) were fewer in number than those who had graduated from secondary school or higher in both groups. Nine patients had fallen in the past 6 months with no such experience among controls. Both groups had frequent diagnoses of schizophrenia, followed by those with bipolar disorder. Comparisons of demographic data for inpatients who fell and controls are shown in Table 1.

**Table 1:** Demographic data of patients who did (n = 80) and did not fall (n = 160)

Variable	Total	Inpatient falls	Controls	P-value
		N (%)	N (%)	
Age, mean (S.D.), y	240	47.37 (12.95)	47.01 (13.19)	np*
		Min–Max = 20–70	Min–Max = 18–70	
Gender				
Male	162	54 (67.5)	108 (67.5)	np†
Female	78	26 (32.5)	52 (32.5)	
Marital status				
Single, separate, divorce	168	61 (76.25)	107 (66.88)	< 0.001†
Couple	72	19 (23.75)	53 (33.13)	
Education level				
≤ primary education	100	32 (40.00)	68 (42.50)	0.08†
≥ Secondary education	140	48 (60.00)	92 (57.50)	
Fallen in past 6 months				
Yes	9	9 (11.2)	0 (0.0)	N/A
No	231	71 (88.8)	160 (100.0)	
Diagnosis, N (%) Major diagnosis based on ICD-10				
1. Schizophrenia	140	42 (52.5)	98 (61.2)	N/A
2. Bipolar disorders	25	8 (10.0)	17 (10.6)	
3. Depressive disorder	12	4 (5.0)	8 (5.0)	
4. Substance abuse	13	3 (3.8)	10 (6.2)	
5. Alcohol dependence	16	3 (3.8)	13 (8.1)	
6. Dementia or Alzheimer's disease	3	2 (2.5)	1 (0.6)	
7. Psychosis due to medical condition	12	8 (10.0)	4 (2.5)	
8. Mental retardation with psychosis	11	8 (10.0)	3 (1.9)	
9. Acute psychosis	3 (1.2)	2 (2.5)	1 (0.6)	
10. Schizophrenia with other psychiatric disorders	5 (2.1)	0 (0.0)	5 (3.1)	

*p-value calculated using paired t-test

^†^p-value calculated using McNemar's Chi-Square test

np refers to non significant at p-value ≤ 0.05

### Fall incident characteristics

The majority of fall incidents were reported in 2014 (27.8%). The day with the highest frequency of falls was Tuesday. Fall incidents occurred more frequently in the ordinary ward than in the special ward and pre-ICU ward. Times of having falls indicate the highest frequency is during the night shift (midnight. - 8.00 am.) (57.5%). Some 55.4% of the patients fell after 2 weeks of hospitalization, and 30.1% fell within the first seven days of hospitalization. Locations of the falls were most likely the bedroom (43.8%) and the bathroom (37.5%). Activities during falls were bathroom-related (42.5%) and getting up from the bed or chair (30%), respectively. The characteristics of incidents of falls are shown in Table 2.

**Table 2:** Characteristics of incidents of falls

Descriptions	Number of falls	%
Year of falls (A.D.)		
2010	8	3.3
2011	52	21.7
2012	60	25.0
2013	54	22.5
2014	66	27.8
Day of week		
Monday	9	11.3
Tuesday	22	27.5
Wednesday	11	13.8
Thursday	14	17.5
Friday	8	10.0
Saturday	8	10.0
Sunday	8	10.0
Time periods of fall incidents		
Daytime (7.00 a.m.–8.00 p.m.)	33	41.3
Nighttime (8.00 p.m.–7.00 a.m.)	47	58.8
Shift work		
Morning shift (8.00 a.m.–16.00 p.m.)	18	22.5
Afternoon shift (16.00 p.m.–24.00 p.m.)	16	20.0
Night shift (24.00 p.m.–8.00 a.m.)	46	57.5
Unit type		
Ordinary ward	53	66.3
Special or extraordinary ward	26	32.5
Pre-ICU	1	1.3
Number of days from admission to fall		
Within 7 days after admission	25	30.1
Between 8 and 14 days after admission	12	14.5
≥ 14 days after admission	46	55.4
Activity during falls		
1. Slipping/falling in bathroom	34	42.5
2. Getting up from bed/chair/wheelchair	24	30.0
3. Walking	16	20.0
4. Running/climbing up	4	5.1
5. Falling down from bed	2	2.5
Location of falls		
1. Bathrooms	35	43.8
2. Bedrooms	30	37.5
3. Walking/outdoor areas	10	12.6
4. Relaxation/dining room	5	6.3
Consciousness before fall		
Alert	66	82.5
Confused	14	17.5
Impact of fall injury		
Level E	69	86.3
Level F	10	12.5
Level G	1	1.3

Level E = resulted in temporary harm to the patient and required intervention

Level F = resulted in temporary harm to the patient and required initial or prolonged hospitalization

Level G = resulted in permanent patient harm

### Univariate analysis of risk factors for inpatient falls

Univariate analysis revealed that inpatients who fall were significantly more likely to have acute psychosis (uOR = 6.88, 95% CI 2.56, 18.53), have a co-occurring physical illness, and use drugs with five or more types of medicines (uOR = 2.97, 95% CI 1.57, 5.63; uOR = 3.91, 95% CI 2.10, 7.29). Univariate analysis also showed that taking an atypical antipsychotic medication or cardiovascular agent significantly increased the risk of a fall (uOR = 2.64, 95% CI 1.48, 4.70; uOR = 2.17, 95% CI 1.05, 4.51 respectively). In contrast, taking a typical psychiatric drug significantly reduced the risk of a fall (uOR = 0.53, 95% CI 0.31, 0.92). Staffing factors that could substantially protect the risk of a fall included the nurse-to-patient ratio and the number of patients in the ward. The results showed that one nurse for 16–30 patients and one nurse for 31–45 patients compared to one nurse for 15 patients decreased risk of fall by 0.28 and 0.17 times respectively (95% CI 0.11, 0.69; 95% CI 0.05, 0.53). As to skill mix, a 1 to 1 proportion of nurses to ULNs on duty marginally significantly increased the risk of fall compared to having one nurse to three ULNs (0.33). However, other risk factors did not substantially affect inpatient falls, as shown in Table 3.

**Table 3:** Univariate and multivariate conditional (fixed-effects) logistic regression of risk factors associated with inpatient falls in a matched case–control study

Variables	Inpatient falls N (%)	Controls N (%)	uOR (95% CI)	P-value	aOR (95% CI)	P-value
*Clinical characteristics*						
Psychiatric condition						
No acute psychosis	60 (75.00)	151 (94.38)	1			
Having acute psychosis	20 (25.00)	9 (5.63)	6.8 (2.56–18.53)	< .001	4.62 (1.14–18.66)	.031*
Number of psychiatric disorders						
1	70 (87.50)	150 (93.75)	1			
2	10 (12.50)	10 (6.25)	2.9 (.84–5.18)	.112	.75 (.16–3.56)	.719
Co-occurring physical illnesses						
0	49 (61.25)	131 (81.88)	1			
1–3	31 (38.75)	29 (18.13)	2.97 (1.57–5.63)	< .001	1.29 (.47–3.52)	.617
Number of drugs used						
1–4	32 (40.00)	113 (70.63)	1			
≥ 5	48 (60.00)	47 (29.38)	3.91 (2.10–7.29)	< .001	2.62 (1.04–6.61)	.040*
*Medication*						
Typical psychiatric drugs						
No	47 (58.75)	69 (43.13)	1			
Yes	33 (41.25)	91 (56.88)	.53 (.31–0.92)	.025	.88 (.30–2.62)	.827
Atypical psychiatric drugs						
No	30 (37.50)	97 (60.63)	1			
Yes	50 (62.50)	63 (39.38)	2.64 (1.48–4.70)	< .001	2.63 (.95–7.32)	.064
Antidepressants						
No	64 (80.00)	131 (81.88)	1			
Yes	16 (20.00)	29 (18.13)	1.14 (.56–2.35)	.712	NI	
Benzodiazepines						
No	14 (17.50)	39 (24.38)	1			
Yes	66 (82.50)	121 (75.63)	1.52 (.77–2.99)	.229	NI	
Anticonvulsants						
No	49 (61.25)	113 (70.63)	1			
Yes	31 (38.75)	47 (29.38)	1.59 (.87–2.90)	.127	.70 (.29–1.69)	.424
Lithiums						
No	77 (96.25)	153 (95.63)	1			
Yes	3 (3.75)	7 (4.38)	0.84 (.20–3.49)	.812	NI	
Antiparkinsons						
No	13 (16.46)	28 (17.61)	1			
Yes	66 (83.54)	131 (82.39)	1.12 (.49–2.56)	.781	NI	
Antidiabetic agents						
No	72 (90.00)	151 (94.38)	1			
Yes	8 (10.00)	9 (5.63)	2.06 (.69–6.15)	.192	NI	
Anticholesterol agents						
No	73 (91.25)	153 (95.63)	1			
Yes	7 (8.75)	7 (4.38)	2.13 (.71–6.43)	.181	NI	
Cardiovascular agents						
No	60 (75.00)	137 (85.63)				
Yes	20 (25.00)	23 (14.38)	2.17 (1.05–4.51)	.037	1.22 (.40–3.71)	.729
*Staffing factors*						
Number of nurses in the shift						
1	35 (43.75)	76 (47.50)				
2–4	45 (56.25)	84 (52.50)	1.23 (0.65–2.33)	.523	NI	
Nurse-to-patient ratio						
1:1–15	20 (25.00)	17 (10.63)	1			
1:16–30	43 (53.75)	96 (60.00)	.28 (.11–.69)	.006	.45 (.12–1.68)	.237
1:31–45	17 (21.25)	47 (29.38)	.17 (.06–.54)	.002	1.05 (.14–7.84)	.962
Number of patients in the ward						
≤ 25	34 (42.50)	53 (33.13)	1			
26–50	22 (27.50)	59 (36.88)	.39 (.16–.95)	.038	.15 (.03-.87)	.034*
≥ 51	24 (30.00)	48 (30.00)	.84 (.37–1.90)	.687	.35 (.04–2.90)	.330
No. of ULNs in the shift						
2	45 (56.25)	94 (58.75)	1			
3–5	35 (43.75)	66 (41.25)	1.16 (0.60–2.23)	.659	NI	
Proportion of skill mix						
.33	1 (1.25)	5 (3.13)				
.50	34 (42.50)	72 (45.00)	3.45 (.30–39.41)	.318	2.27 (.11–47.87)	.598
.67	24 (30.00)	61 (38.13)	2.67 (.23–30.92)	.432	6.31 (.22–181.81)	.283
1.00	21 (26.25)	22 (13.75)	9.10 (.73–113.92)	.087	16.54 (.46–588.42)	.124
Type of unit						
Ordinary ward	53 (66.25)	118 (73.75)	1			
Private ward & ICU	27 (33.75)	42 (26.25)	1.48 (.80–2.72)	.208	NI	

ULNs: unlicensed nurse assistants; NI: not inclusion; uOR: unadjusted odds ratio; aOR: adjusted for all variables in the table p ≤ .15); CI: confidence interval

*p < 0.05

### Multivariate analysis of risk factors for inpatient falls

A multivariate conditional logistic regression model was adjusted for multiple variables associated with inpatient falls p ≤ 0.15, as shown in Table 3. After adjusting for the effect of proportion of skill mix, the final multivariate model showed that inpatients with acute psychosis have a 4.34 times increased risk of falling versus those with no acute psychosis condition (95% CI 1.15, 18.66). Drug use ≥ 5 types of medicines increased the risk of falling 3.06 times versus those who used < 5 medications (95% CI 1.59, 5.88) and those taking atypical psychiatric drugs had a 2.74 times increased risk of falling versus those who did not take any atypical psychiatric drug (95% CI 1.35, 5.58). In contrast, 26–50 patients in a ward reduced the risk of falling by 0.17 compared to smaller wards of ≤ 25 patients (95% CI 0.03, 0.87), as shown in Table 4. The interaction effects among the covariates in the final model were not found. The model was appropriately fitted (AIC = 139.8239, BIC = 167.6691, Hosmer–Lemeshow statistics = 1.05, p = 0.292).

**Table 4:** Multivariate conditional logistic regression model of risk factors associated with inpatient falls in a matched case–control study in a psychiatric hospital

Variables	Adjusted OR	95% CI	P-value
Acute psychosis condition			
No	1		
Yes	4.34	1.45–13.05	.009
Number of drugs used			
1–4	1		
≥ 5	3.06	1.59–5.88	.009
Atypical psychiatric drugs			
No	1		
Yes	2.74	1.35–5.58	.005
Number of patients in the ward			
≤ 25	1		
26–50	.17	.04-.72	.015
≥ 51	.38	.08–1.74	.213

The model is adjusted for acute psychotic condition, number of drugs used, atypical psychiatric drugs, number of patients in the ward, and the proportion of skill mix (.33, .50, .67, 1.00)

AIC = 139.8239; BIC = 167.6691), No interaction between covariates

Model fitted appropriately (Hosmer–Lemeshow statistic = 1.05; p = .292)

## Discussion

Psychiatric inpatient falls were associated with multiple risk factors. Our findings differed from some past evidence and highlight the challenges for improving inpatient service. The results reveal that patient-related factors such as clinical characteristics and medication use, combined with some staffing factors, affect the risk of falling during psychiatric hospitalization.

The frequency of falls increased for each year of the study period. This increase in falls may be explained because this period coincided with improving the hospital system of risk management recording. Fall incidents commonly occur mid-week during the night shift, and there may be more staff per patient on Monday than on other days or at night. Also, there are many therapeutic and administrative activities with an increased patient flow during the week's first day. Before falling, the most common activities were those in the bathroom, followed by getting up from bed/chair/wheelchair in the bedroom. The most frequent activities before falls were different in a report of inpatient fall with injury in the psychiatric unit among veterans. The most frequent activity related to veterans' falls was getting up from a bed, chair or wheelchair (21.3%), with bathroom-related falls at 9% [24]. These falls might be because bathroom floors in general in Southeastern Asia, including Thailand, get wet when people shower or leave something on the floor. Therefore, an analysis of the cause of falls in different cultures is essential for improving fall prevention.

Of clinical characteristics of psychiatric inpatients, mental illness with acute psychosis was most associated with increased risk of fall, followed by a co-occurring physical condition. The findings aligned with the prospective matched case–control study that reported that patients with more severe positive psychiatric symptoms were significantly associated with increased risks for fall and fall-related injury [12, 25, 26]. Also, a lack of medical problem history significantly decreased the risks for fall-related injury [12]. Simultaneously, patients taking polypharmacy or using five or more types of medications had a greater chance of falling. These results are consistent with a study by Cooper and colleagues [27] that polypharmacy, especially concurrent use of two or more psychotropic and psychoactive, may increase the risk of falls in a skilled nursing facility in proportion to the total use of these agents.

Among the types of medication used, this current study found that atypical antipsychotic and cardiovascular agents significantly and independently increase the risk of falls by 2.64 and 2.17 times respectively compared to those who don't use them. Also, the use of typical antipsychotics reduces the risk of falls 0.53 times compared to those not using them. The other psychotropic drugs were not significantly and independently associated with increased inpatient falls. This finding partly supported two previous studies in psychiatric inpatients [3, 8], which showed that atypical antipsychotics and cardiovascular agents affected inpatient falls. In another previous study, Lavse et al. found that any psychotropic medication including atypical antipsychotics, typical antipsychotics, benzodiazepines, selective serotonin reuptake inhibitors (SSRIs), atypical antidepressants, anticonvulsants and mood stabilizers, lithium, and drugs for Alzheimer's disease were independently associated with the risk of falls. Also, cardiovascular agents such as ACE inhibitors, beta-blockers, calcium channel blockers, and alpha-blockers were independently related to the risk of falls [3]. Atypical antipsychotic use was associated with adverse hyperglycemic events, with the co-administration of multiple antipsychotic medicines related to more adverse hyperglycemic events [28]. There was evidence that seizures may be induced by non-ketonic hyperglycemia [29], which possibly leads to falls occurring. Regarding the effect of typical and atypical antipsychotics on risk of fall, this study’s finding showed both agreement and difference with previous studies [10, 11]. A case–control study of nursing home residents reported that both typical and atypical antipsychotic associated with increased hospitalization for femur fracture [11]. A prospective study [10] found that fall events in geriatric inpatients were related to receiving a typical antipsychotic drug (OR = 2.90, 95% CI 1.34, 6.15). A low dose (1 mg/day) of risperidone was associated with decreased falls, especially in patients who exhibited wandering behaviors. In contrast, a 2 mg/day dosage may increase the risk of falls in ambulatory persons with a low level of wandering. Therefore, psychological and behavioral problems, type of drug, dosage, and timing of antipsychotic administration affect fall incidents need to be further investigated.

Regarding staffing factors, the studies of nurse staffing and adverse outcomes in psychiatric units are scarce. Also, the circumstances and the provision of care in psychiatric units are different from medical, surgical, or other departments. However, the previous studies in five separate units in a general hospital found that the association between staffing levels and fall was not linear but was cubic spline or curve-shaped and varied by unit types, especially on step-down or medical units [17]. In these units, there was an association between nurse staffing and falling: at lower staffing levels, the fall rate increased as staffing increased, but at moderate and high staffing levels, the fall rate decreased as staffing increased. The finding from this study also reinforced the previous studies that showed a non-linear relationship between nurse per patient ratio and risk of fall. A nurse per patient ratio of 1:16–30 can lower the risk of fall better than a nurse per patient ratio of 1:31–45 or 1:1–15. Also, the higher total number of nurses in the shift was not significantly associated with the risk of falls. This finding was consistent with a report from the study of inpatient falls in a general hospital, and skilled nursing facilities that a higher number of the nursing staff was not associated with falls occurring [17, 30]. A previous study conducted in a public hospital inpatient psychiatric care environment found that patient falls with injuries were not significantly related to patient-to psychiatric nurse staff ratio but were negatively associated with manager leadership skill [31]. It is possible that rather than the patient-to nurse ratio being of most importance, the quality of nursing training or awareness of the risk of falls in psychiatric inpatients is essential in preventing inpatient falls. The issue needs to be explored in further study. Likewise, 3–5 ULNs in the shift were not significantly associated with falls occurring versus two ULNs in the shift. Also, nurses' skill mix to ULNs with ratios of 0.50 and 1.00 in the shift was not associated with falls occurring versus a mix ratio of 0.33.

The number of patients staying in the ward was not linearly related to the risk of falling. The risk of falls was reduced 0.39 times with 26–50 patients versus ≤ 25 patients in wards. On the other hand, wards with ≥ 51 patients were not significantly associated with falls. Wards with an intermediate number of patients are found in ordinary wards. The standard ward layout is an open room. There are 20–30 beds in the room. Everyone can see each other's movements and daily activities and help each other. Therefore, falls are prevented because they are detected, and assistance is given before a severe fall occurs. In addition, these findings may result because psychiatric inpatients go through various severity phases. Some are in the acute phase, early stability phase, and stability phase. The psychiatric unit's circumstance is to provide managed daily activities and an environment like living at a patient's home. Daily activities are scheduled to encourage the patients to improve their psychosocial functioning. The patients can ambulate around the unit, socialize and help each other. Thus, the crowding of patients in the unit may not be a risk factor for falls. Private wards for VIP patients and pre ICU wards that separate patients with violent behavior for care in a specialty unit were not significantly associated with falls versus ordinary wards.

Finally, the multivariate model revealed that the most significant and strongest predictor for increased risk of fall incidents of psychiatric inpatients was having acute symptoms of psychosis. The second predictor was polypharmacy, taking five or more drugs simultaneously and the third predictor was taking atypical psychiatric drugs. This three identified predictors in the model were intrinsic factors. An organizational element, 26–50 patients in each ward, significantly protected the risk of falls. In contrast, wards with ≥ 51 patients were not significantly associated with falls versus wards with ≤ 25 persons. This finding is consistent with Blair & Grumen’s finding that the total number of geriatric psychiatric inpatients in a ward was inversely related to the number of falls [1]. With low patient numbers, the fall rate increased, while with high patient numbers, the fall rate decreased. The possible reason is that the staff "let down their guard" when they had few patients or were more relaxed and less vigilant [1].

The research findings highlight the essential intrinsic and organizational factors that need to be considered for psychiatric hospitalization fall prevention. According to intrinsic factors, the fall risk assessment tool can detect vital risk factors to preventing falls. Regarding extrinsic factors, hospital staff can improve the structure at the organizational level. Thailand lacks a mental health workforce, and psychiatric units are significantly understaffed compared to the suggested norms of health staffing required for health facilities. According to the annual report of the Department of Mental Health, Ministry of Public Health, Thailand, in 2012, there were 1.11 psychiatrists and 6.19 nurses per 100,000 of population, working in twelve mental health services in the Bangkok area [32]. While in the E.U., there were over 24 nurses per 100,000 population working in mental health [33]. Further studies need to focus on the staffing-related or organizational factors affecting patient outcomes in mental health services. There is no standardized acuity tool available for planning and managing psychiatric staff in mental health units.

### Limitation

This study examined a psychiatric hospital in an upper-middle-income country with a shortage of psychiatric professionals and under healthcare resources. The study used only one data source from a psychiatric hospital, so the results may not be generalizable in other countries with different contexts. Staffing numbers considered in this paper did not exclude the number of staff who joined the hospital or who had meetings out of the unit or those who took the day off for vacation or sickness. Researchers believed that the number of inpatient falls reported in this study was much lower than the actual fall incidence. Previous studies provide reasons for underreporting fall incidences as fellows: first, the negative attitude of staff in reporting fall incidents; second, fall with noninjury is most likely to be unreported; and third, time constraints in filling out the reports of falls [34]. Also, for unwitnessed falls, psychiatric inpatients were less likely to accurately report the fall to staff versus patients with other diagnoses because of altered mental activity [35]. Therefore, the characteristics of falls from the reported incident to the hospital's risk management center in this study mainly included falls with an injury with no information related to any falls reported in the medical reports or patient reports. Although our research was done some time ago, it still reflects the condition in the psychiatric ward in Thailand. It provides one of the first associations among patient and staffing factors with psychiatric inpatient falls in Southeast Asia. Age is a critical indicator in assessing inpatient falls. Age in this study population was not discussed since subjects were age-matched, although patients in 5-year groups may have been such a wide range that some age differences were missed.

## Conclusion

Fall of psychiatric inpatients is a crucial problem but can be preventable. Our findings highlight the patient-related factors for the prevention of falls. Results show acute psychotic condition, polypharmacy, and taking atypical psychiatric drugs associated with increased risk of falls. Among organizational factors, wards with between 26 and 50 patients have significantly fewer falls, an essential protector from falls. Furthermore, researchers need to investigate the dosage of medications such as atypical and typical antipsychotics and cardiovascular agents to see if medicine levels might increase or protect from falls. Also, the timing of typical and atypical antipsychotic drug administration may induce the occurrence of falls. Polypharmacy as risk means a detailed evaluation of drug interactions that may cause psychiatric inpatient falls is warranted. Regarding staffing-related factors, neither R.N. nor ULN ratios or skill mix proportions were significantly associated with inpatient falls. Yet, the necessary ratio of staff to patient and proportion of staff mix in psychiatric units is needed to protect against inpatient falls and should be investigated and verified. Administrators of psychiatric hospitals need to determine workforce needs for utilizing staff availability and achieving satisfactory health outcomes like limiting fall incidents. Therefore, future studies should evaluate an adequate staff and skill mix that balances supply and demand for efficient quality of care to prevent adverse outcomes in psychiatric inpatients. Furthermore, a prospective study is recommended to investigate patient and organizational factors and effects from psychiatric inpatient falls.

## Data Availability

The datasets used and analyzed during the current study are not publicly available due to the confidential of the risk management system of the hospital but are available from the corresponding author on reasonable request.

## Abbreviations

ICUs: Intensive Care Units
CVA: Cerebrovascular Accident
RN: Register Nurse
SSRIs: Selective serotonin reuptake inhibitors
ULNs: Unlicensed Nurse Assistants
